# SARS‐CoV‐2 infection shortly after BNT162b2 vaccination results in high anti‐spike antibody levels in nursing home residents and staff

**DOI:** 10.1002/iid3.525

**Published:** 2021-09-09

**Authors:** Doris Urlaub, Natalie Wolfsdorff, Deniz Durak, Frank Renken, Carsten Watzl

**Affiliations:** ^1^ Department for Immunology Leibniz Research Centre for Working Environment and Human Factors (IfADo) at TU Dortmund Dortmund Germany; ^2^ Dortmund Health Department Dortmund Germany

**Keywords:** anti‐SARS‐CoV‐2 spike antibodies, COVID‐19, SARS‐CoV‐2, vaccine

## Abstract

**Introduction:**

One dose of a coronavirus disease 2019 (COVID‐19) vaccine can elicit high antibody titers in individuals who were previously infected by severe acute respiratory syndrome coronavirus 2 (SARS‐CoV‐2). However, it is unclear how a SARS‐CoV‐2 infection shortly after a first COVID‐19 vaccine dose affects antibody responses.

**Methods:**

Here we investigate residents and staff of a nursing home, where a COVID‐19 outbreak occurred shortly after the first BNT162b2 immunization.

**Results and Conclusions:**

Our data show that individuals who got infected as early as 10 days after their first immunization show antibody levels comparable to fully vaccinated individuals.

## Graphical abstract

It is unclear how a severe acute respiratory syndrome coronavirus 2 infection shortly after a first coronavirus disease 2019 vaccine dose affects antibody responses. Here, we show that individuals who got infected as early as 10 days after their first BNT162b2 immunization show antibody levels comparable to fully vaccinated individuals.

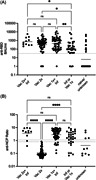

## 1 INTRODUCTION

Several recent studies have demonstrated that a single messenger RNA vaccination in individuals who recovered from coronavirus disease 2019 (COVID‐19) results in high antibody titers that are comparable to noninfected individuals who received two vaccine doses.^1^, ^2^ In Germany, this resulted in the recommendation that individuals who had a documented severe acute respiratory syndrome coronavirus 2 (SARS‐CoV‐2) infection should only receive a single dose of a COVID‐19 vaccine.^3^ In individuals without prior infection, all COVID‐19 vaccines provide already some protection after the first dose.^4^ However, this protection occurs earliest about 14 days after vaccination, and it is not complete.^5^ Therefore, infections shortly after the first dose of a COVID‐19 vaccine are possible. This raises the question, what effect a SARS‐CoV‐2 infection shortly after a first dose of a COVID‐19 vaccine has on antibody titers.

## 2 METHODS

We studied 82 residents and 94 members of the staff of a nursing home who experienced a COVID‐19 outbreak shortly after receiving their first vaccination with BNT162b2 in January 2021. The mean age was 65 years (range: 18–101) and 150 were female (85%). Six weeks after the last infection we analyzed blood samples for anti‐SARS‐CoV‐2 nucleocapsid protein (NCP; EuroImmun) and spike (receptor‐binding domain [RBD]) specific immunoglobulin G antibodies by enzyme‐linked immunosorbent assay (ELISA).^6^ Serum samples were diluted from 1:100 to 1:12,500 and results were expressed as the dilution which still gave the same signal as an internal calibrator of the ELISA, indicating a positive result. The values for samples that were below this detection limit are interpreted as negative and set to 1. The assay was calibrated to the World Health Organization international standard^7^ and values are expressed as binding antibody units (BAU). Antibody titers were compared by using the Kruskal–Wallis test with Dunn's multiple comparison or Mann–Whitney test. The study was approved by the IfADo Ethics Committee (#178).

## 3 RESULTS

**Figure 1:**
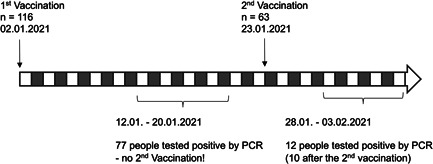
Timeline of vaccinations and COVID‐19 outbreak in a nursing home. First injections of BNT162b2 were given on January 2, 2021. A COVID‐19 outbreak was first detected on January 12. Second vaccinations with BNT162b2 were administered on January 23. From January 28 to February 3 more individuals tested positive for SARS‐CoV‐2 by RT‐PCR. COVID‐19, coronavirus disease 2019; RT‐PCR, reverse transcription‐polymerase chain reaction; SARS‐CoV‐2, severe acute respiratory syndrome coronavirus 2

A total of 116 individuals of a nursing home received their first injection of BNT162b2 on January 2, 2021 (Figure 1). Shortly after, the nursing home faced a COVID‐19 outbreak and between January 12 and 20 seventy‐seven individuals tested positive for SARS‐CoV‐2 by reverse transcription‐polymerase chain reaction (RT‐PCR), fifty‐three of whom had been vaccinated. Among the infected vaccinated individuals 19.5% showed symptoms and four had to be hospitalized. However, the available data about disease severity including pre‐existing conditions were too limited to draw any conclusions about the protective effect of the first vaccine dose. Individuals who tested positive did not received their second dose. On January 23 sixty‐three individuals received a second injection of BNT162b2. From January 28 to February 3 twelve more individuals tested positive for SARS‐CoV‐2 by RT‐PCR, ten of whom had been vaccinated twice.

We first tested for the presence of anti‐SARS‐CoV‐2 NCP antibodies, which are only induced upon SARS‐CoV‐2 infection and not by immunization with BNT162b2. Among the individuals with a documented SARS‐CoV‐2 infection 83.3% had detectable anti‐NCP antibodies and 96.7% had detectable anti‐spike‐RBD antibodies (Figure S1). We found one case of positive anti‐NCP antibodies without a documented infection, which we counted as a case of prior SARS‐CoV‐2 infection.

**Figure 2:**
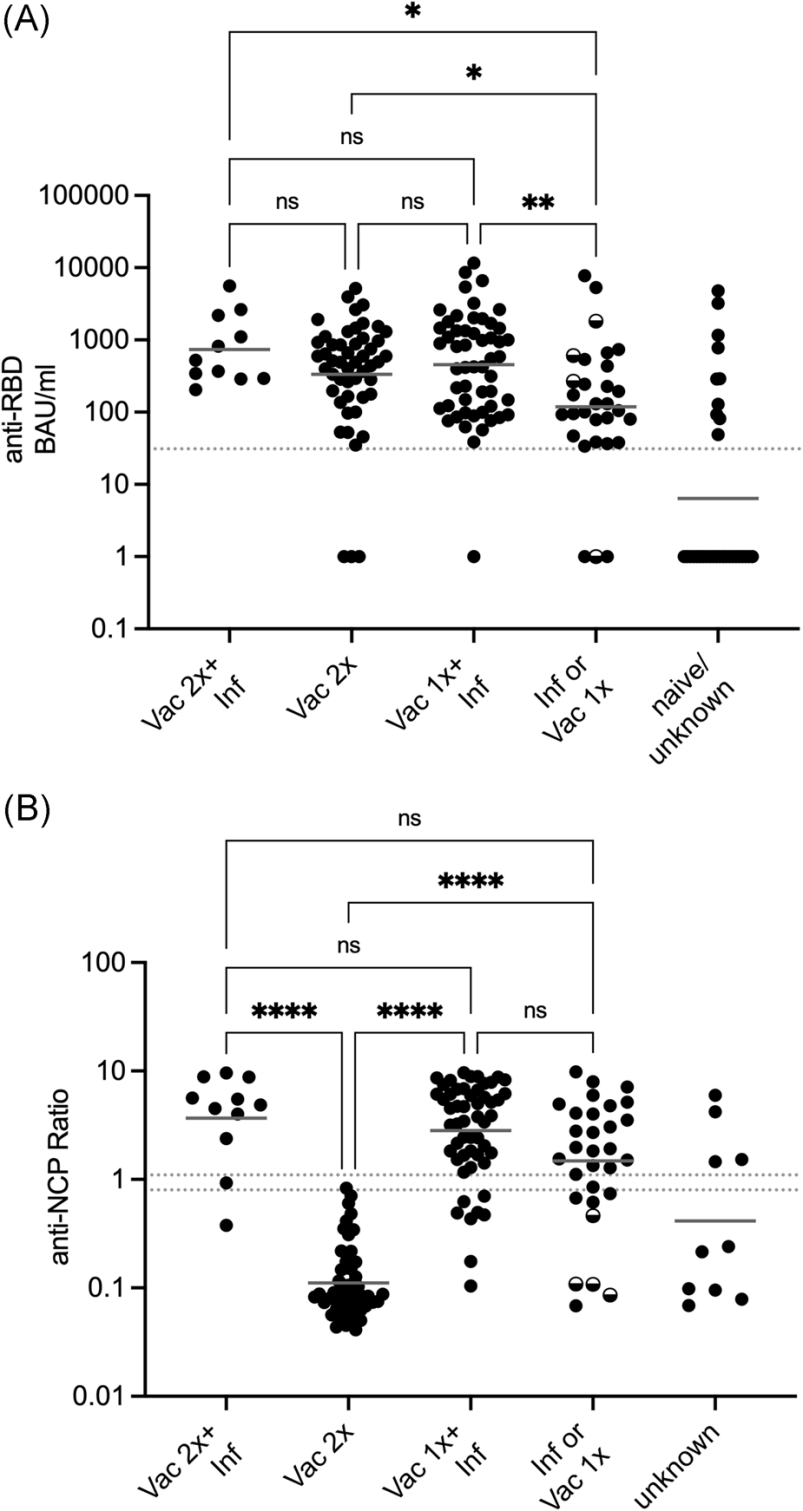
Anti‐SARS‐CoV‐2 spike (RBD) and nucleocapsid protein (NCP) titers in the different groups. Individuals were grouped according to their SARS‐CoV‐2 infection (Inf) or their doses of vaccination (Vac). Half‐filled dots in the “Inf or 1xVac” group represent individuals who were vaccinated once. Antibodies specific for SARS‐CoV‐2 spike‐RBD (expressed as binding antibody units [BAU]/ml) (A) or NCP (anti‐NCP) (B) were determined by ELISA. The assay limit for a positive value is indicated by the dotted line. Groups were compared using Kruskal–Wallis test with Dunn's multiple comparison (adjusted *p* values [A] 2xVac+Inf vs. 1xVac or Inf *p* = .0213, 2xVac vs. 1xVac or Inf *p* = .0268, 1xVac+1xInf vs. 1xVac or Inf *p* = .0076, [B] 2xVac+Inf vs 2xVac *p* < .0001, 2xVac vs. 1xVac+Inf *p* < .0001, 2xVac versus 1xVac or Inf *p* < .0001). ELISA, enzyme‐linked immunosorbent assay; RBD, receptor‐binding domain; SARS‐CoV‐2, severe acute respiratory syndrome coronavirus 2

Among the 32 individuals who did not receive a vaccination and who did not have a SARS‐CoV‐2 infection documented by positive RT‐PCR we found 10 (31.3%, all members of the staff) who tested positive for anti‐spike‐RBD antibodies. Only four of these also had detectable anti‐NCP antibodies (Figure 2). Anti‐spike‐RBD antibody titers were variable and ranged from 49 to 4775 BAU/ml. A total of 26 individuals had a PCR‐confirmed SARS‐CoV‐2 infection without vaccination and we could detect anti‐NCP antibodies in 21 and anti‐spike‐RBD antibodies in 24 of them (92.3%) with a geometric mean titer (GMT) of 119 BAU/ml (95% confidence interval [CI]: 54–262; Figures 2 and S1). Additionally, four individuals only received a single dose of BNT162b2 (two on January 2 and two on January 23) and we found detectable anti‐spike‐RBD antibodies in three of them.

In fully vaccinated individuals we found high anti‐spike‐RBD antibodies with a GMT of 334 BAU/ml (95% CI: 197–568), which was significantly higher than the titer of the infected or single vaccinated group. However, we also found three individuals (5.9%, ages 101, 96, and 89) with no detectable antibody levels after two doses of BNT162b2. More importantly, the 53 individuals who received one dose of BNT162b2 and were then infected with SARS‐CoV‐2 10–18 days later had similar antibody titers (GMT: 453 BAU/ml; 95% CI: 285–720) compared to the group who received two vaccinations. However, one individual of this group had no detectable anti‐spike‐RBD antibodies. Finally, there was a tendency towards higher antibody titers in the individuals who were infected after their second vaccination (GMT: 733 BAU/ml; 95% CI: 355–1515) compared to the fully vaccinated group, but due to the small sample size this difference was not significant. We repeated the analysis for the different groups and compared male and female individuals. We saw a trend that females had higher anti‐spike‐RBD titers in the groups that were vaccinated and infected (Figure S2), although this difference was not significant due to the small number of males in our study. Interestingly, this difference was not observable in the 2x vaccinated group.

**Figure 3:**
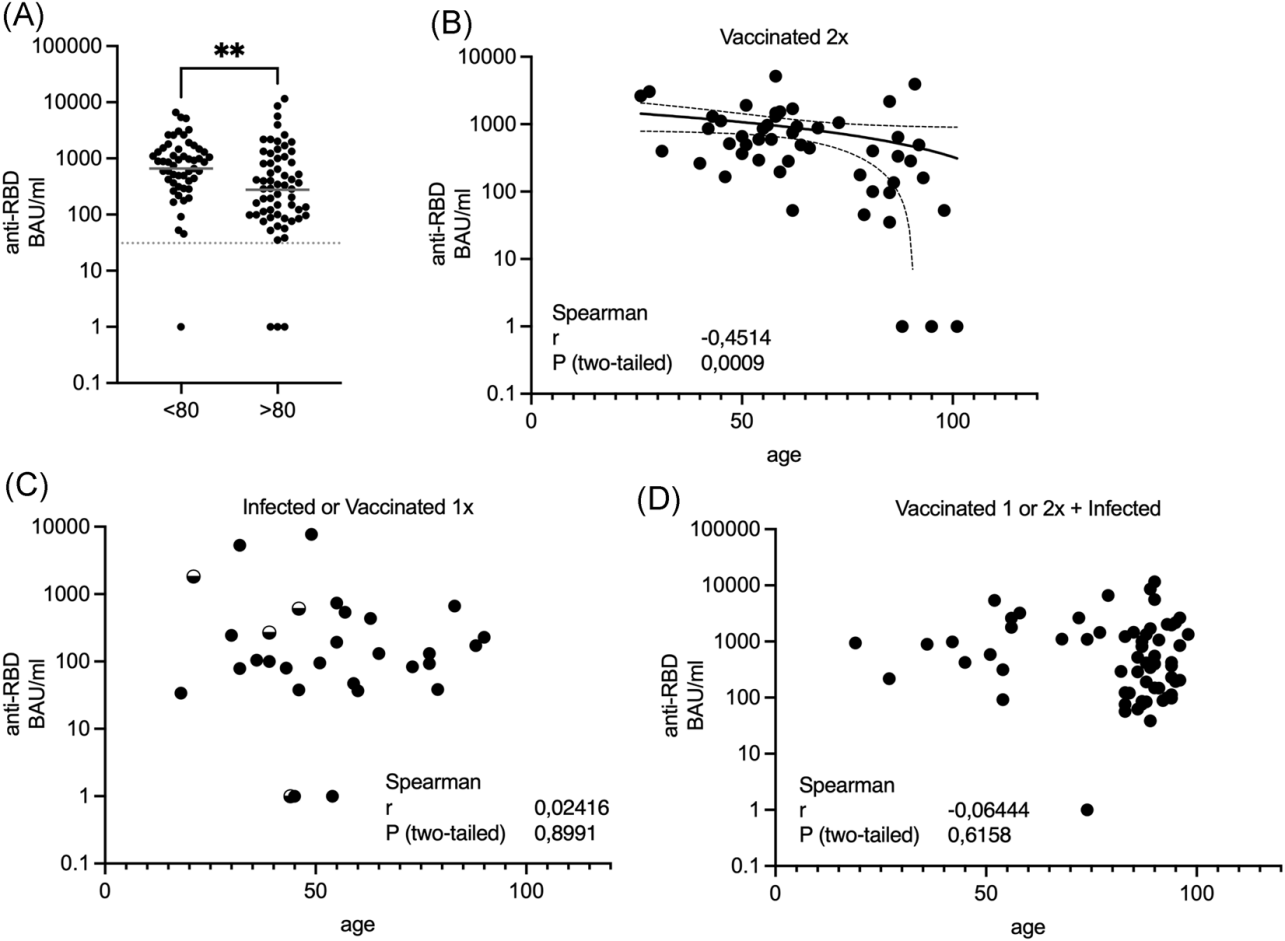
Age‐dependent anti‐spike‐RBD antibody titers. (A) Individuals from the “Vac 2x,” the “Vac 1x+Inf,” or the Vac 2x+Inf” groups described in Figure 2 were grouped according to their age and analyzed for their anti‐spike‐RBD antibody titer. Groups were compared using the two‐tailed Mann–Whitney test (*p* = .0018). (B–D) Correlation analysis of anti‐spike‐RBD titers in the “Vac 2x” (B), the “Inf or 1xVac” group (C), or the “Vac 1x+Inf” and “Vac 2x+Inf” group (D) as described in Figure 2 in relation to age. (B) Linear regression with the equation “Y = −14.94 × X + 1820” and 95% CI of −29.58 to −0.2961 for the slope and 821.8–2818 for the *Y*‐intercept is shown. BAU, binding antibody units; CI, confidence interval; RBD, receptor‐binding domain

As we tested residents and staff, we wanted to test if age has an influence on antibody titers in individuals who received two vaccinations or who were infected after their vaccination. We observed a significant difference between individuals less than 80 years (GMT: 661 BAU/ml; 95% CI: 449–972) compared to more than 80 years (GMT: 278 BAU/ml; 95% CI: 171–449, two‐tailed Mann–Whitney test *p* = .0018; Figure 3A). Using Spearman's rank correlation, we found a significant negative correlation between age and anti‐spike‐RBD antibody titers among the individuals that received two vaccine doses (Figure 3B). Interestingly, this correlation was no longer observable when we analyzed individuals who had been infected with SARS‐CoV‐2 with or without immunization (Figure 3C,D).

## 4 DISCUSSION

In the COVID‐19 outbreak investigated here, we identified 10 individuals who had anti‐spike‐RBD antibodies, four of which also had anti‐NCP antibodies and who had therefore been infected without having tested positive by PCR. All these individuals were staff members ranging from 22 to 62 years, demonstrating the difficulty of identifying all (asymptomatically) infected individuals during an outbreak. Our data also show that two individual triggers of an anti‐SARS‐CoV‐2 immune response are necessary to result in high antibody titers. However, these triggers can originate from two doses of BNT162b2 or from one immunization and one infection, even in the elderly and even if this infection happens as early as 10 days after the immunization. This was surprising, as the effectiveness of a second vaccine dose can be impaired by administering it too close to the first one. Finally, three triggers, resulting from an infection after two doses of BNT162b2, only showed a nonsignificant trend towards higher antibody titers.

Our data also confirm that BNT162b2 antibody titers are affected by age.^8^ Interestingly, this effect was only significant after 2x vaccination. When individuals were additionally infected, there was no clear effect of age, suggesting that the antibody response upon infection is not significantly influenced by age. We also found an effect by sex, as males tended to have lower anti‐spike‐RBD antibody titers, but only when analyzing individuals who had been infected with or without 1x vaccination. We saw no sex difference in the 2x vaccine group. This suggests that in contrast to age, the antibody response upon infection is affected by sex.^9^ Interestingly, we found nonresponders who did not develop any detectable anti‐spike‐RBD antibodies after two doses of BNT162b2, similar to recent reports.^10^ Therefore, it may be necessary to confirm the success of COVID‐19 immunizations in the elderly.

## CONFLICT OF INTERESTS

The authors declare that there are no conflict of interests.

## AUTHOR CONTRIBUTIONS

*Conception and design of project*: Frank Renken and Carsten Watzl. *Data acquisition and analysis*: Doris Urlaub, Natalie Wolfsdorff, and Deniz Durak. *Interpretation of data*: Doris Urlaub and Carsten Watzl. *Writing of manuscript*: Carsten Watzl.

## Supporting information

Supplementary information.

## Data Availability

The data that support the findings of this study are available from the corresponding author upon reasonable request.
