# Supplementary material for: SARS‐CoV‐2 infection shortly after BNT162b2 vaccination results in high anti‐spike antibody levels in nursing home residents and staff
Source: Immun Inflamm Dis. 2021 Sep 9;9(4):1702–6. doi: 10.1002/iid3.525 (PMC8589355; doi:10.1002/iid3.525)
Supplement: Supplementary file 1 — Supplementary information. [file IID3-9-1702-s001.pdf]

Supplemental information

SARS-CoV-2 Infection shortly after BNT162b2 Vaccination results in high anti-Spike Antibody Levels in Nursing Home Residents and Staff.

Doris Urlaub[1], Natalie Wolfsdorff[1], Deniz Durak[2], Frank Renken[2], Carsten Watzl[1]

[1] Department for Immunology, Leibniz Research Centre for Working Environment and Human Factors (IfADo) at TU Dortmund, Dortmund, Germany.

[2] Dortmund Health Department, Dortmund, Germany

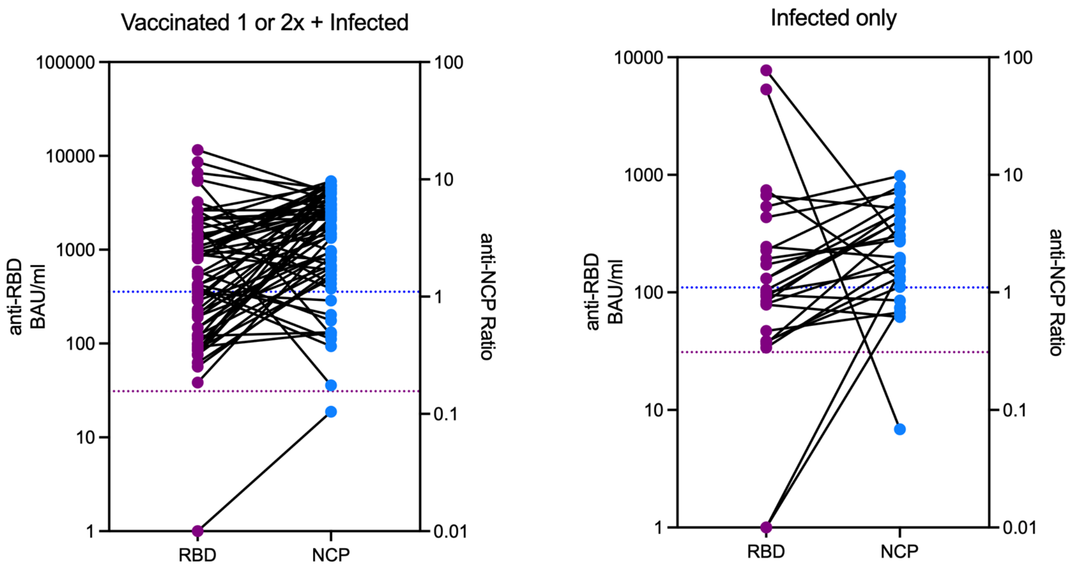

Figure S1: Comparison of anti-SARS-CoV-2 spike (RBD) and anti-nucleocapsid protein (NCP) titers in the different groups. Antibodies specific for SARS-CoV-2 spike-RBD (expressed as Binding Antibody Units (BAU)/ml) (shown in purple) or NCP (shown in blue) from the indicated groups described in figure 2 were determined by ELISA. Connected dots are from the same individual. The assay limit for a positive value is indicated by the dotted line (purple for anti-RBD and blue for anti-NCP).

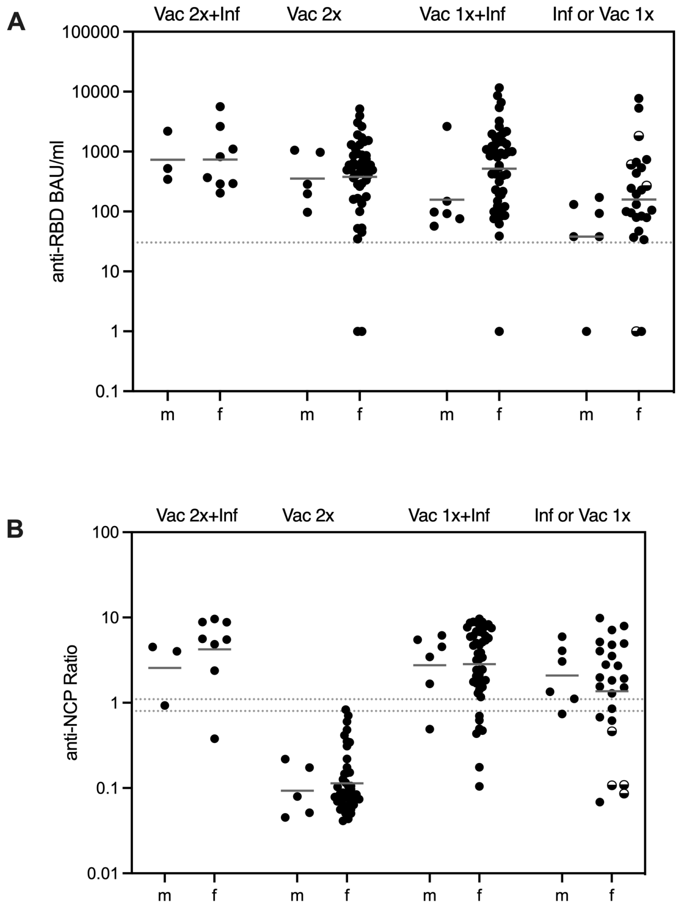

Figure S2: Anti-SARS-CoV-2 spike (RBD) and NCP titers in the different groups. Individuals were grouped as described in figure 2 and sub-divided in male (m) and female (f). Antibodies specific for SARS-CoV-2 spike-RBD (expressed as Binding Antibody Units (BAU)/ml) (A) or nucleocapsid protein (anti-NCP) (B) were determined by ELISA. Lower detection limit is indicated by the dotted line. Due to the small group size, no statistical analysis was performed.
